# Patients in long-term maintenance therapy for drug use in Italy: analysis of some parameters of social integration and serological status for infectious diseases in a cohort of 1091 patients

**DOI:** 10.1186/1471-2458-6-216

**Published:** 2006-08-23

**Authors:** Gianluca Quaglio, Fabio Lugoboni, Cristian Pattaro, GICS, Linda Montanari, Alessandro Lechi, Paolo Mezzelani, Don C Des Jarlais

**Affiliations:** 1 Medical Service for Addictive Disorders, Department of Internal Medicine, University of Verona, Italy; 2 Unit of Epidemiology and Medical Statistics, Department of Medicine and Public Health, University of Verona, Italy; 3 Unit of Genetic Epidemiology, Institute of Genetic Medicine, EURAC Research, Bolzano/Bozen, Italy; 4 Scientific Intercentres Collaborative Drug Users Group (GICS), Italy; 5 European Monitoring Centre for Drugs and Drug Addiction (EMCDDA), Lisbon, Portugal; 6 Department of Internal Medicine, University of Verona, Italy; 7 Edmond de Rothschild Foundation Chemical Dependency Institute, Beth Israel Medical Center, New York City, USA

## Abstract

**Background:**

Heroin addiction often severely disrupts normal social functioning. The aims of this multi-centre study of heroin users in long-term replacement treatment were: i) to provide information on aspects of social condition such as employment, educational background, living status, partner status and any history of drug addiction for partners, comparing these data with that of the general population; ii) to assess the prevalence of hepatitis, syphilis and HIV, because serological status could be a reflection of the social conditions of patients undergoing replacement treatment for drug addiction; iii) to analyse possible relationships between social conditions and serological status.

**Methods:**

A cross-sectional study was carried out in sixteen National Health Service Drug Addiction Units in northern Italy. The data were collected from February 1, 2002 to August 31, 2002. Recruitment eligibility was: maintenance treatment with methadone or buprenorphine, treatment for the previous six months, and at least 18 years of age. In the centres involved in the study no specific criteria or regulations were established concerning the duration of replacement therapy. Participants underwent a face-to-face interview.

**Results:**

The conditions of 1091 drug treatment patients were evaluated. The mean duration of drug use was 14.5 years. Duration was shorter in females, in subjects with a higher educational background, and in stable relationships. Most (68%) had completed middle school (11–14 years of age). Seventy-nine percent were employed and 16% were unemployed. Fifty percent lived with their parents, 34% with a partner and 14% alone. Males lived more frequently with their parents (55%), and females more frequently with a partner (60%). Sixty-seven percent of male patients with a stable relationship had a partner who had never used heroin. HCV prevalence was 72%, HBV antibodies were detected in 42% of patients, while 30% had been vaccinated; 12.5% of subjects were HIV positive and 1.5% were positive for TPHA.

**Conclusion:**

A significant percentage of heroin users in treatment for opiate addiction in the cohort study have characteristics which indicate reasonable integration within broader society. We posit that the combination of effective treatment and a setting of economic prosperity may enhance the social integration of patients with a history of heroin use.

## Background

There is a tendency, not confined to popular opinion, to regard illicit drug users (DUs) as hopelessly enmeshed in their addiction and utterly without prospects for betterment [1]. Heroin addiction often severely disrupts normal social functioning and often leads to severe social stigmatization [2]. The frequent alternation between states of drug induced euphoria and withdrawal, and the time, effort and money needed to obtain the drug makes it quite difficult to maintain employment and satisfactory social relationships. The goals of addiction treatment therefore usually include not only reducing/eliminating illicit drug use but also re-integrating or integrating the addict into society. While there is substantial literature indicating the effectiveness of drug abuse treatment in improving the social functioning of addicts [2-4], there also appears to be the public perception that drug abuse treatment is severely limited in integrating former drug addicts into society at large [3,5].

The aims of this multi-centre study of heroin users in long-term replacement treatment in northern Italy were: i) to provide information on aspects of social integration such as employment, educational background, living status (living with whom), partner status and any history of drug addiction of partners; ii) to assess the prevalence of hepatitis B virus (HBV), hepatitis C virus (HCV), human immunodeficiency virus (HIV) and syphilis, because serological status needs to be considered in relation to the potential social integration of drug treatment patients; iii) to analyse possible relationships between social conditions and serological status. Numerous studies have been carried out in order to analyse the effectiveness of long-term maintenance treatment for heroin users [6-8]. The approach of this study was to collect available data on patients actively undergoing replacement treatment. The aim was to assess the social conditions of a large cohort of patients undergoing drug replacement treatment, compared to the general population in northern Italy. Data are presented for patients in maintenance treatment for at least six months. The study was carried out in the Veneto Region, in the north of Italy, where the unemployment rate in 2001 was 4% and 9.5% of the population are graduates. In 1999, the per capita gross domestic product (GDP) in this area was € 20,286, higher than the average for Italy as a whole € 17,086 [9].

## Methods

### Population

The data presented in this cross-sectional study were collected from February 1, 2002 to August 31, 2002, in 16 National Health Service Drug Addiction Units (NHS-DAUs). All NHS-DAUs contributing data were part of a Regional Scientific Research Group (GICS in the Italian acronym) dealing with drug problems. These publicly funded NHS-DAUs provide counselling, treatment for drug withdrawal, agonist and antagonist therapy, medical care, and psychological therapy, as described elsewhere [10]. NHS-DAUs only treat outpatients. Recruitment eligibility was based on three criteria: i) maintenance treatment with methadone or buprenorphine; ii) treatment for the previous six months; iii) at least 18 years of age. In the centres involved in the study, the clinical approach of replacement therapy was to achieve successful ongoing maintenance rather than abstinence. In these centres no specific criteria or regulations were established concerning the duration of replacement therapy. Eligible and consenting participants underwent standardised face-to-face interviews carried out by doctors and nurses in each centre. Participation in the study was voluntary and anonymous. No incentive of any kind was provided for participation. The majority of those declining to be interviewed stated that they did not have the 20–30 minutes required to complete the questionnaire. Questions regarding current behaviour (employment, educational background, living status, partner status, history of drug addiction of the partner and serological status for infectious diseases) referred to the six-month period prior to the interview. Written and signed informed consent was obtained. The study has been approved by the local Ethical Committee.

The classification of employment and unemployment was consistent with the criteria of the European Union, which uses the principles of the International Labour Office and which the Italian Institute of Statistics (ISTAT) applies to data concerning the general population [11]. The definition of an employed person is someone of at least fifteen years of age with one of the following characteristics: 1) regular employment even if no work was done during the reference week; 2) paid work of at least one hour during the reference week. The classification of an unemployed person was met by subjects with one of the following characteristics: 1) they said they were looking for employment; 2) they had actively sought employment in the 4-week period prior to the interview; 3) they were willing to immediately take employment, if offered. The International Standard Classification of Education was used for the classification of educational status. Vocational status was considered separately.

### Laboratory analyses

Serological testing was performed at 16 different laboratories. The analysis of serological status was part of a scheduled entry medical examination. For determination of anti-Hepatitis C Virus, Cobas Core HCV EIA II (Roche Laboratories, Mannheim, Germany) was used. Human immunodeficiency virus antibodies were assessed by enzyme-linked immunosorbent assay (ELISA; Biotest, Germany) and confirmed with Western blot (DiaSorin, Saluggia, Italy); for the determination of anti-Hepatitis B Virus markers (anti-HBs, total anti-HBc, HBs-Ag), all laboratories used Cobas Core EIA test kits (Roche Laboratories, Mannheim, Germany); to determine markers for syphilis, the Treponema pallidum haemoagglutination test (TPHA) was used. All labs used standard methods specified by the test kit manufacturers.

### Statistical analysis

Chi-squared and Fisher's exact tests were used to assess relationships between categorical variables. Univariate and multivariate association of various factors with the duration of drug use was assessed using the Kruskal-Wallis test and a linear regression model, respectively. The goodness-of-fit of the linear model was assessed through standard diagnostics. To avoid biased estimates, we chose to leave age out of the model. The data from the subjects in drug abuse treatment were compared, wherever possible, with data for the general population in northern Italy [9,11,12]. To ensure the consistency of the comparison with the general population, which has a slightly different age distribution, the direct standardisation method was used [13]. To assess the association of social integration variables with serological variables, the following logistic regression models were fitted (one for each serological variable): logit (θ) = α + β_1,1..15 _(recruitment site) + β_2 _(age) + β_3 _(sex) + β_4 _(duration of drug use) + β_5,1..3 _(education) + β_6,1..2 _(employment status) + β_7,1..3 _(living status) + β_8,1..2 _(partner status) + β_9,1.2 _(partner's use of heroin) + ε where: θ was the probability of HIV, HBV, HCV and TPHA, respectively, and ε was a random error following a binomial distribution [13]. All the independent variables but age were included as factors. Due to co-linearity between "partner's use of heroin" and "partner status", the two variables were combined: "steady relationship with a non drug user partner", "steady relationship with a drug user", "no steady relationship or no partner". The le Cessie-van Houwelingen normal test statistic was used to assess the goodness-of-fit of the model [14]. Statistical analyses and data management were carried out using R 2.2.1. [15].

## Results

### Baseline characteristics

During the study period, 1759 patients were under treatment in 16 centres (NHS-DAU). Of the total, 530 were not eligible because they had been in replacement therapy for less than six months, or were treated with naltrexone or other forms of therapy for opiate drugs, or were being treated for addiction to substances other than heroin. Of the 1229 eligible subjects, 138 (11.2%) did not agree to take the test and 1091 (88.8%) underwent standardised interviews. Of the interviewees, 920 (84%) were males and 171 (16%) were females. The mean age was 33.0 years (SD: 6.4 years), 50% were between 25 and 35 years old. The most common therapy was methadone (88%); these patients were, on average, older than patients receiving buprenorphine (p = .002) See Table 1 for a detailed view of social characteristics.

### Drug use characteristics

A large majority (89%), reported injecting as their principal route of heroin administration before starting treatment, and 11% reported intranasal use. The youngest patients (age<35 years old) more frequently sniffed heroin than older subjects (13.2% vs 7.9%, p < 0.006). The mean duration of drug use was 14.5 years (SD = 6.8). A differential duration of drug use was observed for buprenorphine DUs vs methadone DUs (medians were 12 and 14, respectively, Kruskal-Wallis test p = 0.0073), educational levels (median for elementary school DUs was 16, 14 for middle school DUs, 12 for vocational school DUs and 12 for secondary or higher school DUs; p < 0.0001), living status (median was 12, 16, 16, 10 for DUs living with parents, a partner, alone or friends, respectively; p < 0.0001). No differences were observed for gender (p = 0.2150) and employment status (p = 0.0912). The correlation between duration and age was 0.7 8 (95%CI: 0.75–0.80). The selected model included the recruitment site, gender, therapy, educational level, employment status, living status and partner status. The fitting proved reasonable, with normally distributed residuals and limited heteroscedasticity (see Additional file 1). Results are reported in Table 2. Males used heroin for a longer time than females (estimated regression coefficient b = 1.51, 95%CI: 0.42, 2.60). The higher the educational level the lower the duration of drug use (b = -2.10, 95%CI: (-3.59, -0.61), b = -3.37, 95%CI: (-5.17, -1.56); b = -4.23, 95%CI: (-6.03, -2.43) for middle, vocational and secondary or higher school level, respectively, versus elementary school). With reference to living status, living with a partner (b = 4.85, 95%CI: 3.57,6.13) and living alone (b = 2.25, 95%CI: 1.08,3.53) are associated with increased drug use duration. Subjects in stable relationships (married or unmarried) had a shorter duration drug use than subjects without a stable partner (b = -2.75, 95%CI: (-3.85, -1.65) and b = -2.23, 95%CI: (-3.87, -0.59), respectively) (Table 2).

### Educational status

**Figure 1:**
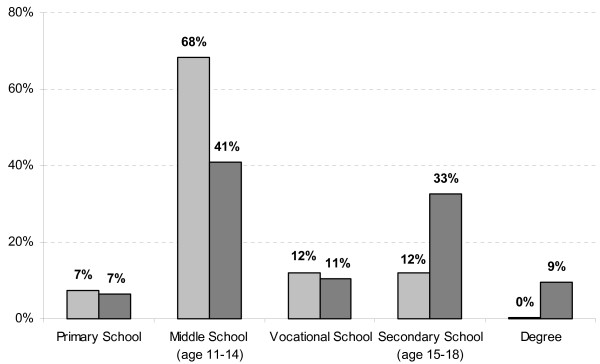
Educational background of DUs compared to the general population in northern Italy (DUs in grey, general population in black).

Seven percent of subjects had attended only primary school, 68% attended until the school-leaving age in Italy, and 12% had finished secondary school. Females had higher educational levels than males (p = 0.001, Table 1). Compared to the 15–49 year old population of northern Italy, the cohort of patients had a lower educational background (p < .001): in fact they were concentrated in the lowest level of education (7% in the elementary school and 68% in the middle school vs 7% and 41%, respectively, in the general population). Only 12% of patient DUs had finished secondary school and almost none had a degree vs 33% and 9%, respectively, in the general population (see Figure 1).

### Employment status

**Figure 2:**
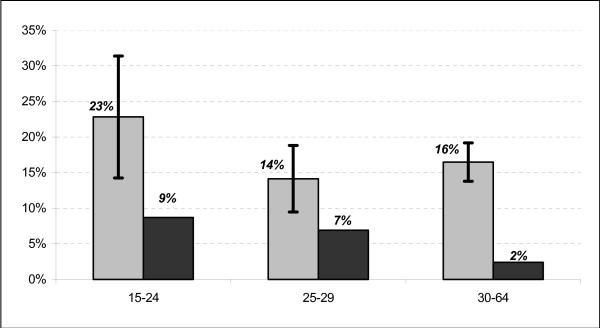
Percentage of DUs unemployed, compared to people of the same age in the general population of northern Italy (DUs in grey, general population in black).

Seventy-nine percent of subjects were employed and 16% were unemployed. Figure 2 compares unemployment rates, stratified by age, for the cohort and the general population in northern Italy. The unemployment rate within the study group was higher than in the general population (p < 0.01): the difference was 14% for 15–24 age group, 7% for 25–29 age group and 14% for 30–64 age group, respectively.

### Living status

**Figure 3:**
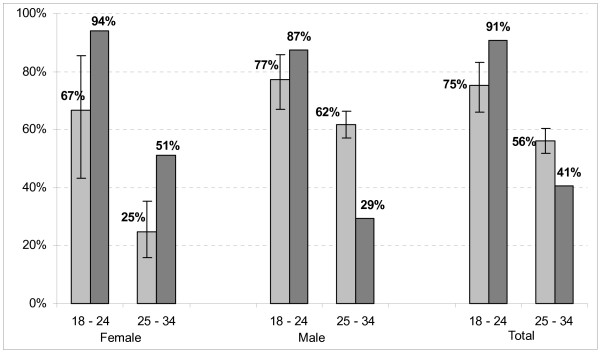
Percentage of DUs living with parents compared to subjects of the same age in the general population in northern Italy (DUs in grey, general population in black).

Of the total sample, 50% of patients lived with their parents, 34% with partners and 14% alone. Males lived with their parents more frequently than females (55% vs 24%), and females were more frequently living with a partner (60% vs 29%, p-value for difference in distribution <0.001, Table 1). When restricted to 18–34 year-old DUs (to allow comparison with northern Italy population data [9]), the percentage of DUs living with parents was significantly smaller than the general population when younger individuals were considered (18–24), while it was bigger when older subjects (25–34) were observed (Figure 3, column 5 and 6). Moreover, when stratifying by gender, additional differences were observed. The percentage of females DUs living with parents was lower than the general population (p < 0.05) both for the 18–24 and the 25–34 year-old age groups for women (Figure 3, column 1 and 2). On the other hand, males DUs over 25 years of age were more often living with parents than males in the general population (p < 0.05) (Figure 3, column 4).

### Partner status

**Figure 4:**
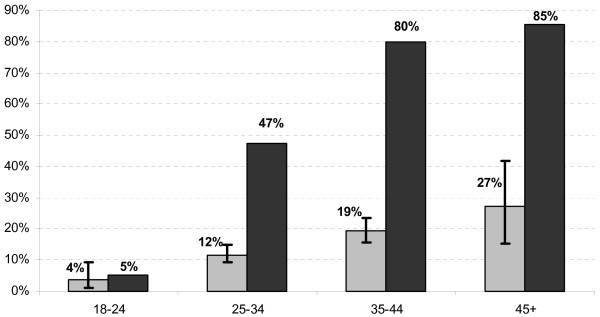
Percentage of married subjects (and 95%CIs) by age, compared to the general population in northern Italy (DUs in grey, general population in black).

Fifty-one percent of patients said they had a stable relationship. Females were more likely to report being in a stable partnership than males (77% vs 46%, p < 0.001, Table 1); 14.4% of all patients were married (157/1091), slightly more females (32/171, or 18.7%) than males (125/920, or 13.6%) (Table 1). Figure 4 compares the percentage of married patients with the general population in northern Italy: the percentage of married subjects is strikingly lower than the general population at all the ages except for the 18–24 group (p < 0.001).

### History of drug use of the partner

For patients in a stable relationship the history of partners' drug use was investigated: 43% of patients said they had a partner with a history of, or current, drug addiction. Heroin use by sexual partners varied by gender: 33% of males had a partner with personal history of drug addiction (current or in the past), while 23% of female patients had a partner who had never used heroin (p < 0.001, Table 1).

### Number of children

The female patients reported an average of 0.56 (95%CI: 0.44,0.69) children, significantly lower than the birth rate in the Veneto Region, which in 1998–2000 was 1.2 children per woman between 15 and 49 years of age.

### Serological markers

HCV antibodies were detected in 72% of patients; HBV antibodies were detected in 42%, while 30% had been vaccinated. HIV prevalence was 12.5% and 1.5% of patients were positive for TPHA (see Table 3).

### Social characteristics and serological status

The goodness-of-fit for logistic models for HIV (z = 1.58, p = 0.11) and for HCV (z = -0.80, p = 0.42) was satisfying. In the model for HBV and for the TPHA the adjustment for recruitment centre was finally omitted because of the sparseness of data, due to the reduced number of cases. The statistics for the goodness-of-fit supported the validity of the models (z = -1.02, p = 0.31 for HBV and z = -1.75 with p = 0.08 for TPHA). The Odds Ratios (ORs) are reported in Table 4. The main evidence is the strong association of the duration of the drug use with all the serological variables: the OR for a one year increase ranged from 1.13 to 1.19 and was always statistically significant. Subject not employed had a higher risk of having HIV (OR = 1.86, 95%CI: (1.13, 3.05) for the unemployed and OR = 3.52, 95%CI: (1.63, 7.61) for subject which other classifications). Unemployed had also a higher risk of having TPHA (OR = 3.73, 95%CI: 1.26, 11.03). Patients with a steady relationship with a drug user had a higher risk of HCV than subjects with stable relationships with a non drug user (OR = 1.97, 95%CI: 1.16, 3.36). Males had lower rates of TPHA than females (OR = 0.22, 95%CI: 0.07, 0.74).

## Discussion

The study shows a reasonable degree of social integration for subjects, with a substantial percentage in employment, a large majority living with parents or sexual partners, and a majority in stable sexual relationships. Although prevalence for HCV remains high, the percentage of HIV positive patients is considerably lower than ten years ago effectiveness [16]; a relevant percentage of subjects had been vaccinated for HBV and the prevalence for syphilis is extremely low, around 1%.

The educational background of the cohort was lower than the general population in northern Italy. Available literature enabled us to compare the educational level of the cohort with the general population which was aged from 15 years and not from 18. The slight difference of three years does not itself explain the wide difference in the educational level. Compared to males, a higher percentage of female patients had a secondary or higher level of education. Educational background seems to be a protecting factor, in the sense that the higher the educational level the lower the duration of drug use.

Employment has been a traditional measure of drug treatment outcome, and maintaining or improving patient levels of employment reflects treatment effectiveness [17,18]. Although there is always a certain percentage of patients who, for various psychological, sociological or physical reasons, are "unemployable", the employment rate in this group is substantial, with higher unemployment levels than the general population in the same geographical area (Figure 2). Seventy-nine percent of this study sample were employed compared to 50% of persons treated in NHS-DAUs in Italy [19,20]. For the study cohort no data are available for employment prior to the study, so no comparison was carried out. Nonetheless, it is the general opinion of the personnel in treatment centres that patients have higher levels of employment today than in the past. This could be the result among other possible factors of the different approach to substitutive treatment (methadone and buprenorphine) compared to the past, with higher doses and easier access to it, for example by supplying one or more weeks of methadone take-home doses. Maintenance treatment has become easier, without interruption in cases of positivity after urine samples, and increasing the dose of methadone in cases of craving. Therapeutic schedules are customized and detoxification, if considered appropriate, is carried out in a planned fashion. Clinicians routinely reduce the frequency of centre visits for patients who improve and prescribe sufficient quantities of medication to cover the increased time intervals between visits. In Italy the treatment is totally free of charge for the patients. Whether these elements, which certainly helped to provide access to, and the implementation of, treatment, also helped patients to find work is not certain and could be investigated further.

Heroin users, women in particular, leave their families earlier than people of the same age in the general population. As the age increases, men tend to stay at home more than the men in the general population of the same age. This was different for females, for whose only one every four lives with her parents. In view of the fact that 77% of female DUs had a stable partner, (versus only the 46% of males), this could mean females are better able to create stable relationships, irrespective of being DUs. On the other hand, for older males, it seems that family of origin is the principle source of stability and integration. Problems associated with drug use involve the family; the role of the family is therefore important in therapy. In this regard NHS-DAUs frequently involve the families in treatment, with medication administered at home, family support during detoxification programmes, etc. More than half of patients (51%) said they had a stable relationship: of them, the 28% were married.

There were very strong gender differences in terms of partners with a history of addiction; 77% of female heroin users reported a stable relationship with partners with a history of addiction, while only 33% of males reported having partners with histories of addiction. These findings are similar to other studies [21,22]. There is evidence that the influence of the partner on drug treatment may differ among males and females. Some studies have shown that males are more likely to seek treatment due to pressure from their families and/or partners [23], while women receive less support from partners and relatives [24]. Drug addiction is often more damaging for women than for men, due to concurrent problems relating to prostitution and violence in intimate relationships. Lack of emotional support in relationship with partners and having DUs in one's social network fosters relapse and lack of compliance with treatment programs [25]. It may be helpful to provide coordinated treatment to couples where both have a history of addiction: this could be particularly important for female DUs.

The high percentage of subjects who were in stable relationships with partners without histories of heroin addiction, if on one hand is a sign of gradual integration into social life, on the other hand raises the issue of possible transmission of blood-borne viruses from the DUs to their sexual partners [26]. It has been reported that a partner in a "risk category" is more dangerous than "safe sex" with many partners who are not in a risk category [27]. Previous research has shown that condoms are less likely to be used in stable relationships than in occasional or non-cohabiting relationships [28]. It has been reported that 28% of males and 70% of females who become HIV positive have no identifiable risk factor [29]. Recently there has been a change in the pattern of HIV infection in Italy, with a gradual decrease of the epidemic among DUs, and an increase among women who are not substance abusers [30]. This confirms the importance of including the social network and partners of DUs in strategies for the prevention of infectious diseases.

Infectious diseases must be considered an important aspect of the potential social integration of patients. In fact these diseases can cause very serious illness, penalizing employment potential and general health if not treated and followed up. In addition, the diseases can be transmitted to others, potentially restricting interpersonal and sexual relations. Twelve percent of the group was positive for HIV, a substantial reduction from 26% ten years ago [16]. The percentage is higher for females than males (17% vs 12%), as in the country and in other countries as a whole: in Vancouver the percentages are females 35.2% vs males 25.8% [31], and in Catalonia-Spain 42.1% for females vs 35.9% for males [3,32]. This could be the consequence of the higher sexual risk of women DUs, as demonstrated by the higher rate of syphilis (Table 4). Unemployment and higher age were risk factors for HIV, whilst a rich educational background seems to act as a protection. In our opinion, the reduction in prevalence, although partly due to the death of many infected patients in recent years [10,33], reflects changes in lifestyles and the implementation of risk reduction policies. Young DUs, for example, inhale significantly more than older DUs. HCV seroprevalence is high, more than 70%, increasing with the time of drug use, making this the most prevalent infection. Nevertheless, prevalence was lower among patients under 25 years of age (35%): for this reason among young adult DUs there is enough time between starting drug use and HCV infection to target them for prevention [34], even in a population with a very high prevalence for HCV such as that of the present study. A moderate percentage of the patients had been exposed to HBV. Thirty per cent of the cohort were vaccinated against HBV, the highest percentage for heroin drug users in Europe, as reported in literature [3,35,36]. HBV vaccination among heroin users proved feasible and effective when integrated into the regular functioning of drug abuse treatment programs [37,38]. The level of syphilis infection was low, as previous reported [39]. In recent years, a large number of sex workers have arrived in Italy from other countries [40]. This has led to the gradual reduction of participation in commercial sex work by Italian drug users. The percentage of Italian nationals among sex workers in Italy has fallen from over 80% at the beginning of the 90s to less than 10% currently [41,42]. In northern Italy, syphilis is associated with commercial sex work but not with drug use [39]. Unemployment seems to increase the risk of blood-borne and sexually transmitted diseases.

## Conclusion

The patients in this study show a reasonable level of social integration. We would suggest that two factors could be primarily responsible for this level of integration. First, these subjects receive effective long-term maintenance treatment for their addiction. Most receive substitutive treatment in a more flexible and customized manner than in the past. A harm reduction approach by health operators places the emphasis on reducing the negative consequences of drug use, with the goal of gradually limiting these consequences. This contrasts with the rehabilitation approach that favours abstinence as the primary goal of drug treatment. Harm reduction advocates acknowledgement that although many DUs may never achieve total abstinence, they can improve their physical health and social functioning [43]. Secondly, northern Italy has a low unemployment rate (4%) and is economically productive (mean annual per capita GDP: € 20,286). The demand for labour undoubtedly leads to the hiring of more people in drug abuse treatment than would occur in areas with higher unemployment rates. Heroin addiction and other drug abuse problems clearly can occur in societies with all varieties of economic conditions, but relative economic prosperity may increase the likelihood of the social integration of heroin users who enter treatment.

Several study limitations should be acknowledged. Firstly, there was no data on the pre-treatment conditions of these patients. Secondly, the sample contained only patients who had been in treatment for at least six months, although the exact duration of treatment was not known. Thirdly, a number of patients left the programme prematurely, often under unfavourable circumstances. Forth, behaviour data were self-reported and potentially subject to a social desirability bias. The interviewers were highly experienced in working with DUs, however, and research has generally shown that self-reports from DUs are valid [44]. Finally, the study contains a non-random sample of patients from a non-random sample of centres.

In most European countries, between 70–75% of the money spent on the drug problem goes into the criminal justice system and the remainder into social and healthcare programs. There is substantial room for devoting more resources for a better treatment of DUs [3]; this, combined with a favourable economical setting in the society, may enhance social integration of persons with history of heroin use.

## Competing interests

The author(s) declare that they have no competing interests.

## Authors' contributions

Study concept and design: GLQ, FL, PM. Acquisition of data: GICS investigators, CP. Study supervision: FL, CP, DDJ. Analysis and interpretation: GLQ, FL, AL, LM. Drafting of the manuscript: GLQ, PM, FL, DDJ. Statistical expertise: CP. Critical revision: PM, FL, LM, AL. All authors read and approved the final manuscript.

## Pre-publication history

The pre-publication history for this paper can be accessed here:



## Supplementary Material

Additional File 1 Graphical diagnostics for the linear regression model. Diagnostic plotsfor the linear regression model which was used to assess the effect of recruitment site, sex, therapy, educational level, employment status, living status and partner status on the duration of drug use. Click here for file

## Figures and Tables

**Table 1:** Characteristics of the 1091 DUs by gender

**Characteristics**		**Females**	**Males**	**Total**	
		**No. (%)**	**No. (%)**	**No. (%)**	p*
**Age**	< 25	21 (12)	84 (9)	105 (10)	0.194
	25–35	85 (50)	459 (50)	544 (50)	
	35–45	62 (36)	332 (36)	394 (36)	
	>45	3 (2)	45 (5)	48 (4)	
**Therapy**	Methadone	154 (90)	806 (88)	960 (88)	0.365
	Buprenorphine	17 (10)	114 (12)	131 (12)	
**Years of drug use**	< 5	21 (12)	51 (5)	72 (7)	0.016
	5–10	27 (16)	174 (19)	201 (18)	
	10–15	44 (26)	260 (28)	304 (28)	
	15–20	36 (21)	187 (20)	223 (20)	
	20–25	35 (20)	170 (19)	205 (19)	
	≥25	8 (5)	78 (9)	86 (8)	
**Drug assumption**	Intravenous	151 (88)	819 (89)	970 (89)	0.784
	Sniffing	20 (12)	101 (11)	121 (11)	
**Education**	Primary	6 (3)	74 (8)	80 (7)	<.001**
	Middle	106 (62)	638 (69)	744 (68)	
	Vocational	22 (13)	109 (12)	131 (12)	
	Secondary or higher	37 (21)	99 (10)	136 (12)	
**Employment status**	Employed	123 (72)	743 (81)	866 (79)	<.001**
	*Continuously^†^*	*100 (81)*	*645 (87)*	*745 (86)*	
	*Discontinuously^‡^*	*23 (19)*	*98 (13)*	*121 (14)*	
	Unemployed	31 (18)	141 (15)	172 (16)	
	Other	17 (10)	36 (4)	53 (5)	
	*Housewives*	*10 (59)*	*2 (6)*	*12 (23)*	
	*Students*	*4 (23)*	*6 (17)*	*10 (19)*	
	*Invalids-Retired*	*3 (18)*	*28 (78)*	*31 (58)*	
**Living status**	Parents	41 (24)	504 (55)	545 (50)	<.001**
	Partner	103 (60)	267 (29)	370 (34)	
	Alone	24 (14)	133 (14)	157 (14)	
	Friends	3 (2)	16 (2)	19 (2)	
**Partner status**	No steady partner	40 (23)	499 (54)	539 (49)	<.001**
	A steady partner	131 (77)	421 (46)	552 (51)	
	*Unmarried*	*99 (76)*	*296 (70)*	*395 (72)*	
	*Married*	*32 (24)*	*125 (30)*	*157 (28)*	
**Partner's drug use^§^**	No	30 (23)	284 (67)	314 (57)	<.001
	Yes	101 (77)	136 (33)	237 (43)	

**Total**		171 (16)	920 (84)	1091 (100)	

Percentages by column; *chi-squared test p-value for the association between gender and each single variable.

** Fisher's exact test p-value; ^†^Continuously: declaring to be employed; ^‡^Discontinuously: declaring to work but not continuously;^§ ^Percentages are referred to subjects having a steady partner.

**Table 2:** Results of the regression analysis for the duration of drug use

**Characteristics**		**Coeff. †**	**95%CI ‡**	p-value*
**Recruitment site **(ref.: centre no. 1)	centre no. 2	2.14	(-0.92, 5.21)	0.1703
	centre no. 3	-2.29	(-4.44, -0.13)	0.0376
	centre no. 4	1.35	(-1.05, 3.74)	0.2696
	centre no. 5	3.33	(1.36, 5.31)	0.0010
	centre no. 6	2.11	(-0.63, 4.86)	0.1315
	centre no. 7	1.17	(-1.92, 4.26)	0.4569
	centre no. 8	2.60	(-0.12, 5.32)	0.0607
	centre no. 9	-0.25	(-2.34, 1.85)	0.8163
	centre no. 10	-1.88	(-4.86, 1.10)	0.2169
	centre no. 11	-1.76	(-4.96, 1.45)	0.2825
	centre no. 12	0.50	(-2.89, 3.90)	0.7702
	centre no. 13	-0.50	(-2.46, 1.46)	0.6166
	centre no. 14	-1.13	(-4.17, 1.91)	0.4655
	centre no. 15	0.78	(-1.58, 3.13)	0.5176
	centre no. 16	1.75	(-0.18, 3.69)	0.0751
**Sex **(Ref.: Females)	Males	1.51	(0.42, 2.60)	0.0068
**Therapy **(ref: Methadone)	Buprenorphine	-1.35	(-2.73, 0.03)	0.0557
**Education **(ref: Primary)	Middle	-2.10	(-3.59, -0.61)	0.0058
	Vocational	-3.37	(-5.17, -1.56)	0.0003
	Secondary or higher	-4.23	(-6.03, -2.43)	<0.0001
**Employment **(ref: Employed)	Unemployed	0.48	(-0.59, 1.55)	0.3780
	Other	0.21	(-1.58, 1.99)	0.8210
**Living status **(ref: Parents)	Partner	4.85	(3.57, 6.13)	<0,0001
	Alone	2.25	(1.08, 3.53)	0,0002
	Friends	-1.62	(-4.54, 1.31)	0.2783
**Partner **(ref: No steady partner)	Steady and unmarried	-2.75	(-3.85, -1.65)	<0,0001
	Steady and married	-2.23	(-3.87, -0.59)	0.0076

† Coeff.: regression coefficient; ‡ 95% Confidence Interval; * p-value for the significance of the single factor.

**Table 3:** Serology of 1091 DUs by gender

		**No. of subjects (%)**			
		
**Serology**		Female*	Male*	Total*	p*
**HIV**	Negative	138 (80.7)	761 (82.7)	899 (82.4)	0.040
	Positive	29 (17.0)	107 (11.6)	136 (12.5)	
	Unknown	4 (2.3)	52 (5.6)	56 (5.1)	
**HBV**	Negative	47 (27.5)	195 (21.2)	242 (22.1)	0.096
	Positive	76 (44.4)	385 (41.8)	461 (42.2)	
	Unknown	6 (3.5)	55 (6.0)	61 (6.0)	
	Vaccinated	42 (24.6)	285 (31.0)	327 (30.0)	
**HCV**	Negative	33 (19.3)	209 (22.7)	242 (22.2)	0.806
	Positive	127 (74.3)	657 (71.4)	784 (71.9)	
	Unknown	11 (6.4)	54 (5.9)	65 (6.0)	
**TPHA**	Negative	128 (74.9)	679 (73.8)	807 (74.0)	0.060
	Positive	6 (3.5)	11 (1.2)	17 (1.5)	
	Unknown	37 (21.6)	230 (25.0)	267 (24.5)	

Percentages by column; *p-value of the chi-square test for association.

**Table 4:** Association between serological status and social-integration conditions**

**Characteristics**		**HIV**		**HBV**		**HCV**		**TPHA**	
		**OR**	95%CI*	**OR**	95%CI*	**OR**	95%CI*	**OR**	95%CI*
**Centre**	centre no. 1	1				1			
	centre no. 2	1.71	(0.37,7.99)	-	-	6.63	(1.26,34.98)	-	-
	centre no. 3	1.85	(0.54, 6.27)	-	-	2.52	(1.09,5.85)	-	-
	centre no. 4	1.73	(0.49, 6.07)	-	-	13.23	(2.73,64.17)	-	-
	centre no. 5	1.44	(0.48, 4.33)	-	-	3.74	(1.54,9.06)	-	-
	centre no. 6	1.74	(0.44, 6.97)	-	-	6.88	(1.72,27.56)	-	-
	centre no. 7	1.26	(0.25, 6.39)	-	-	-	-	-	-
	centre no. 8	0.94	(0.22, 3.99)	-	-	1.93	(0.67,5.62)	-	-
	centre no. 9	1.53	(0.47, 5.01)	-	-	2.93	(1.25,6.83)	-	-
	centre no. 10	3.84	(0.89, 16.53)	-	-	1.13	(0.36,3.62)	-	-
	centre no. 11	-	-	-	-	1.41	(0.44,4.52)	-	-
	centre no. 12	2.91	(0.64, 13.25)	-	-	11.69	(1.31,104.25)	-	-
	centre no. 13	1.05	(0.33, 3.41)	-	-	2.06	(0.99, 4.29)	-	-
	centre no. 14	0.65	(0.06, 6.45)	-	-	2.49	(0.77, 8.08)	-	-
	centre no. 15	0.61	(0.14, 2.64)	-	-	1.58	(0.65, 3.86)	-	-
	centre no. 16	0.85	(0.27, 2.65)	-	-	0.77	(0.37, 1.58)	-	-
**Age**	one year increase	1.01	(0.95, 1.07)	1.01	(0.97, 1.05)	0.99	(0.95, 1.03)	1.00	(0.87, 1.15)
**Sex**	Female	1		1		1		1	
	Male	0.64	(0.37, 1.11)	1.14	(0.70, 1.87)	0.75	(0.44, 1.29)	0.22	(0.07, 0.74)†
**Duration of the drug use**	one year increase	1.14	(1.08, 1.21)†	1.13	(1.09, 1.18)†	1.19	(1.14,1.24)†	1.14	(1.00, 1.31)†
**Education**	Primary	1		1		1		1	
	Middle	0.68	(0.35, 1.31)	1.18	(0.66, 2.11)	1.83	(0.92, 3.61)	0.91	(0.18, 4.72)
	Vocational	0.42	(0.16, 1.07)	0.95	(0.44, 2.06)	1.24	(0.56, 2.75)	1.17	(0.16, 8.70)
	Secondary or higher	0.48	(0.19, 1.20)	0.57	(0.27, 1.22)	1.26	(0.56, 2.83)	0.51	(0.04, 6.44)
**Employment**	Employed	1		1		1		1	
	Unemployed	1.86	(1.13, 3.05)†	1.62	(0.98, 2.67)	1.62	(0.92, 2.85)	3.73	(1.26,11.03)†
	Other	3.52	(1.63, 7.61)†	0.92	(0.37, 2.24)	0.94	(0.56, 1.58)	1.47	(0.16, 8.70)
**Living status**	Parents	1		1		1		1	
	Partner	1.52	(0.69, 3.38)	1.18	(0.66, 2.11)	0.94	(0.56, 1.58)	0.52	(0.10, 2.61)
	Alone	1.05	(0.56, 1.96)	0.88	(0.52, 1.48)	1.67	(0.39, 5.66)	-	-
	Friends	0.58	(0.06, 5.25)	1.59	(0.43, 5.86)	1.48	(0.93, 2.98)	0.86	(0.20, 3.75)
**Partner**	Steady, non D.U.	1		1		1		1	
	Steady and D.U.	1.80	(0.98, 3.32)	1.28	(0.76, 2.17)	1.97	(1.16,3.36)†	0.23	(0.04, 1.21)
	No partner	1.73	(0.79, 3.80)	1.32	(0.77, 2.26)	1.38	(0.86, 2.23)	0.43	(0.10, 1.92)

**: Logistic regression adjusted by recruitment unit, sex, age and duration of drug use; due to sparseness, the adjustment for recruitment site was omitted for HBV and TPHA; *: 95% Confidence Interval; †: p-value < 0.05.
