# Supplementary material for: Patients in long-term maintenance therapy for drug use in Italy: analysis of some parameters of social integration and serological status for infectious diseases in a cohort of 1091 patients
Source: BMC Public Health. 2006 Aug 23;6:216. doi: 10.1186/1471-2458-6-216 (PMC1570141; doi:10.1186/1471-2458-6-216)
Supplement: Supplementary file 1 [file 1471-2458-6-216-S1.doc]

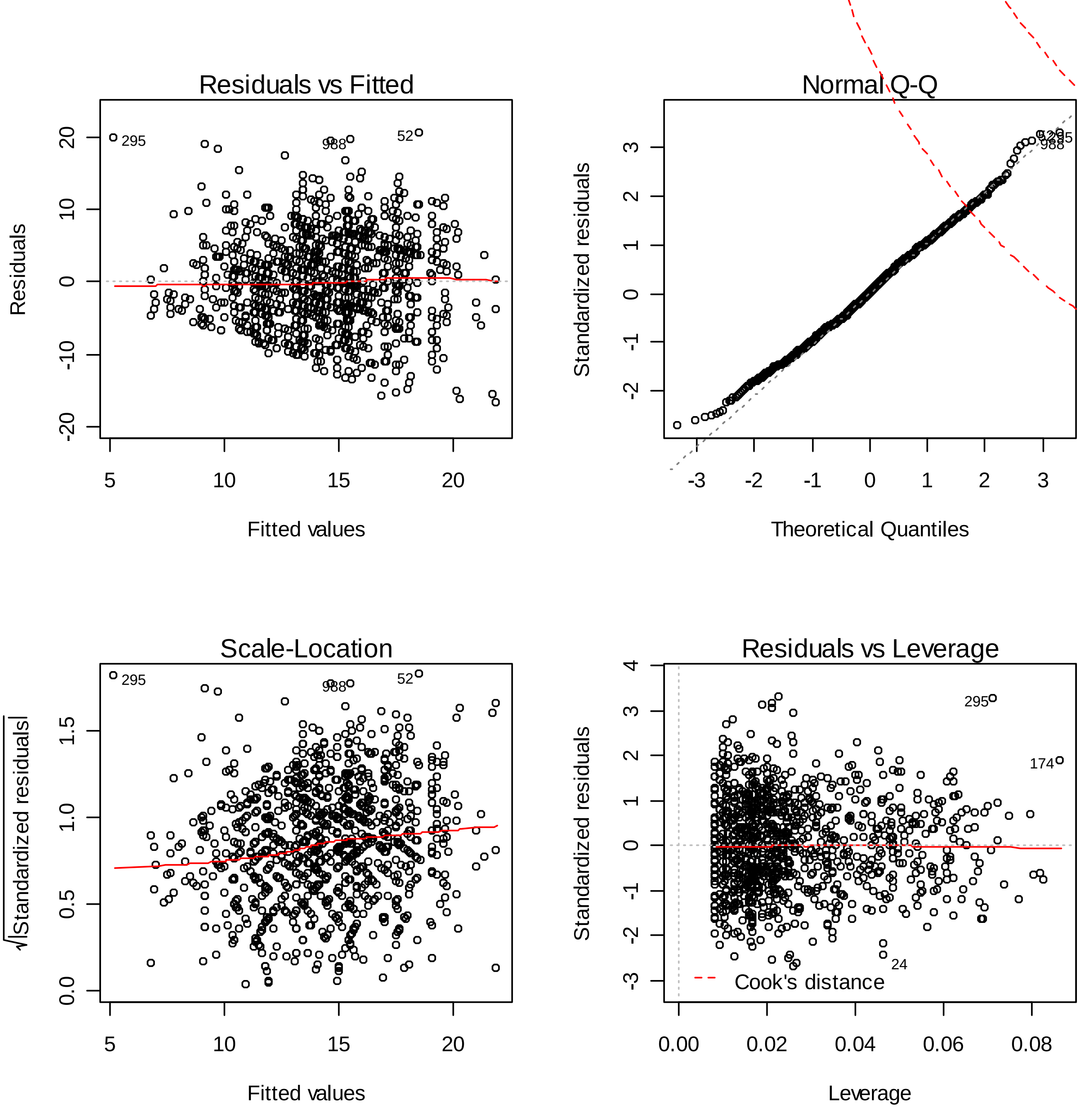
Residuals vs Fitted
Residuals
Fitted values
295
988
52
Normal Q-Q
Standardized residuals
Theoretical Quantiles
52
295
988
Scale-Location
√|Standardized residuals|
Fitted values
295
988
52
Residuals vs Leverage
Standardized residuals
Leverage
295
174
24
Cook's distance
